# Rehabilitation Is the Main Topic in Virtual and Augmented Reality and Physical Activity Research: A Bibliometric Analysis

**DOI:** 10.3390/s23062987

**Published:** 2023-03-09

**Authors:** Angel Denche-Zamorano, Yeray Rodriguez-Redondo, Sabina Barrios-Fernandez, María Mendoza-Muñoz, Antonio Castillo-Paredes, Jorge Rojo-Ramos, Miguel Angel Garcia-Gordillo, Jose Carmelo Adsuar

**Affiliations:** 1 Promoting a Healthy Society Research Group (PHeSO), Faculty of Sport Sciences, University of Extremadura, 10003 Caceres, Spain; 2 Social Impact and Innovation in Health (InHEALTH), University of Extremadura, 06810 Mérida, Spain; 3 Occupation, Participation, Sustainability and Quality of Life (Ability Research Group), Nursing and Occupational Therapy College, University of Extremadura, 10003 Caceres, Spain; 4 Research Group on Physical and Health Literacy and Health-Related Quality of Life (PHYQOL), Faculty of Sport Sciences, University of Extremadura, 10003 Caceres, Spain; 5 Departamento de Desporto e Sauúde, Escola de Sauúde e Desenvolvimento Humano, Universidade de Eúvora, 7004-516 Eúvora, Portugal; 6 Grupo AFySE, Investigación en Actividad Física y Salud Escolar, Escuela de Pedagogía en Educación Física, Facultad de Educación, Universidad de Las Américas, Santiago 8370040, Chile; 7 Physical Activity for Education, Performance and Health, Faculty of Sport Sciences, University of Extremadura, 10003 Caceres, Spain; 8 Universidad Autónoma de Chile, Talca 3467987, Chile

**Keywords:** scientometrics, training, rehabilitation, physical fitness, physical exercise

## Abstract

Researchers’ interest in finding practical applications for virtual reality (VR) and augmented reality (AR) technologies has increased as new devices have become cheaper and more accessible, being used in entertainment, healthcare, and rehabilitation fields, among others. This study aims to provide an overview of the current state of scientific literature related to VR, AR, and physical activity (PA). A bibliometric analysis of studies published between 1994 and 2022 was conducted using The Web of Science (WoS), applying the traditional bibliometric laws and using the VOSviewer software for data and metadata processing. The results revealed an exponential increase in scientific production between 2009 and 2021 (R^2^ = 94%). The United States (USA) was the country/region with the most relevant co-authorship networks (72 papers); the most prolific author was Kerstin Witte, and the most prominent was Richard Kulpa. The most productive journal’s core was composed of high-impact and open access journals. A great thematic diversity was found according to the most used keywords by the co-authors, highlighting concepts such as rehabilitation, cognition, training, and obesity. Then, the research related to this topic is in an exponential development phase, with great interest in the rehabilitation and sports sciences fields.

## 1. Introduction

Virtual reality (VR) and augmented reality (AR) technologies engage a trillion-dollar industry, expected to keep growing in future years [1], attracting companies and investors worldwide [2]. The number of mobile AR device users has increased in recent years, from an estimated 200 million in 2015 to almost one billion users by 2022 [3]. On the one hand, VR enables the user to explore and manipulate real or artificial computer-generated three-dimensional multimedia sensory environments in real time [4]. VR is classified as non-immersive VR, which creates computer-generated virtual environments, with users being aware and controlling their physical environment; these systems are experienced on a screen or videogame console, with keyboards, mice, and/or controllers being used as input devices [5]. Semi-immersive VR generates partially virtual environments where users perceive who is in a different reality focusing on the digital image, while maintaining the link with its physical environment; the immersion is achieved using sensors, displaying powerful graphics, although sometimes, digital media are combined with replicas or physical environments to enhance the immersion [3]. Lastly, immersive VR offers a more realistic simulation experience with image and sound; it requires VR glasses that often split the screen, creating a stereoscopic 3D effect, combined with an input track to establish a believable experience [6]. On the other hand, AR [7] offers an experience which allows for the application of virtual elements in the real world [8].

Both VR and AR are becoming increasingly popular and accessible to users and practitioners, and new forms of utilities are emerging [9]. In addition to the entertainment area, VR and AR applications are transforming healthcare [10], medicine [11], rehabilitation [12,13,14,15], and patient education [16] by providing new and innovative solutions to long-standing challenges. VR is being used in medical education to simulate surgeries and other procedures in a safe and controlled environment, allowing students to gain hands-on experience and build confidence before working on real patients [17]. AR is being used to help patients with physical rehabilitation, allowing them to perform virtual exercises and track their progress in real-time [18]. For instance, a study found that AR-assisted therapy can improve upper limb function in stroke survivors, leading to faster recovery and a reduction in rehabilitation time [19]. VR and AR are also being used to manage pain and anxiety in patients, including dental phobia [20] or helping to manage pain and anxiety levels in patients undergoing medical procedures [21]. Moreover, immersive environments provide instant feedback on users’ actions, generating propitious settings for motor learning achievements [22,23,24,25,26]. VR-based games generate controlled environments that are not easily controllable and quantifiable in the real world, offering the possibility to assess and enhance cognitive and motor rehabilitation [22]. These technologies are also useful in behavioural treatments, users’ education, or follow-up [27]. Moreover, VR has shown some benefits in pathologies such as Parkinson’s disease [12,28,29,30], dementia [31,32], and stroke [33]. VR has also been used in the ageing processes [34,35], providing a balanced interaction between sensorimotor function and cognitive demands [22], being used for balance, fall prevention [36], and kinesiophobia, as it provides gradual exposure to physical challenges [37]. Thus, stroke survivors experienced benefits related to enjoyment and engagement, compared to the treadmill as well as in psychiatric symptomatology [38]. Patients attending cardiac rehabilitation services [39,40,41] and with pulmonary diseases [42,43] reached more positive outcomes, together with other treatments, with these technologies being complementary tools for physical training that improve motivation.

Furthermore, these technologies promote physical activity (PA), increasing their popularity during the COVID-19 confinement, when PA could not be performed collectively or outdoors [44]. VR and AR play an important role in healthy habits behaviour promotion [45,46], with physical inactivity being a risk factor for non-communicable diseases worldwide [47,48,49]. Then, outdoor PA practice may be affected by environmental and social factors [50], so indoor or home-based alternatives could be provided [51], with VR and AR technologies offering the opportunity and motivation to engage in physical exercise [48,52,53]. Some AR and VR applications have demonstrated positive outcomes regarding the PA level [54,55]. For this purpose, VR has been used in healthy individuals [48] and in those with pathologies [39,56,57,58,59], as well as with different age groups [54,60], as active video games produce an energy expenditure equivalent to mild to moderate intensity PA [46].

Although bibliometric studies on RV- and RA-related publications exist in the scientific literature [10,61,62,63], to our knowledge, no scientific mapping of the scientific literature related to VR, VA, and PA has been performed. This study is the first to analyse research trends and new topics of interest to researchers in this field, considering the annual publications trend, the most prolific and cited journals, the most productive and prominent authors, the most cited articles, and the most used keywords by authors, with these being the objectives of this study.

## 2. Materials and Methods

### 2.1. Design and Data

A descriptive bibliometric analysis was conducted to map the scientific research published on VR, AR, and PA in journals indexed in the Web of Science (WoS), the most widely used source for bibliometric analysis, given the large number of journals indexed and the complete information provided on the publications: co-authors, titles, abstracts, keywords, sources, publishers, affiliations, citations, etc. [64,65,66,67]. Thus, a search was carried out on 5 September 2022 in the WoS Core Collection database, with the following search vector: (ti = (“virtual reality”) or ti = (“augmented reality”)) and (ti = (sport) or ti = (sports) or ti = (“physical exercise”) or ti = (“physical training”) or ti = (“physical fitness”) or ti = (“physical activit*”) or ab = (sport) or ab = (sports) or ab = (“physical exercise”) or ab = (“physical training”) or ab = (“physical fitness”) or ab = (“physical activit*”) or ak = (sport) or ak = (sports) or ak = (“physical exercise”) or ak = (“physical training”) or ak = (“physical fitness”) or ak = (“physical activit*”)); restricted to: (1) The Science Citation Index Expanded (SCI-Expanded), The Social Sciences Citation Index (SSCI), and The Emerging Sources Citation Index (ESCI) editions; and (2) to primary research and reviews. The tags used for the searches were TI (title searches), AB (abstract searches), and AK (author keywords search). Data were extracted from The WoS in .xslx and plain text formats and analysed using Microsoft^®^ Excel^®^ for Microsoft 365 MSO version 2206 and the VoSViewer software.

### 2.2. Data Analysis

A descriptive analysis of the annual publications was performed, checking their trend and whether they were in an exponential growth phase analysed with the coefficient of determination (R^2^), using DeSolla Price’s law of Exponential Growth of Science [68,69]. The WoS analyse reports tool was used to conduct a descriptive analysis of the subject categories in which the papers were included. Bradford’s law of science concentration was applied to highlight the most productive journals and those accumulating the highest number of citations [70,71,72]. Lotka’s law was applied to identify the most productive co-authors [73]. The Hirsch index (h-index) was used to identify the most prominent co-authors, with the most productive being those with the highest number of citations among the analysed papers [74,75]. The most relevant contributions were considered to be those with h or the highest number of citations, based on the h-index [75,76]. The most relevant author keywords for co-authors were highlighted by applying Zipf’s law to the keywords used in the whole set of studies [77]. Finally, the VoSViewer software was used to generate visualization graphs on the associations between journals, co-authors, countries/regions, papers, and keywords.

## 3. Results

### 3.1. Annual Publications Trend

**Figure 1:**
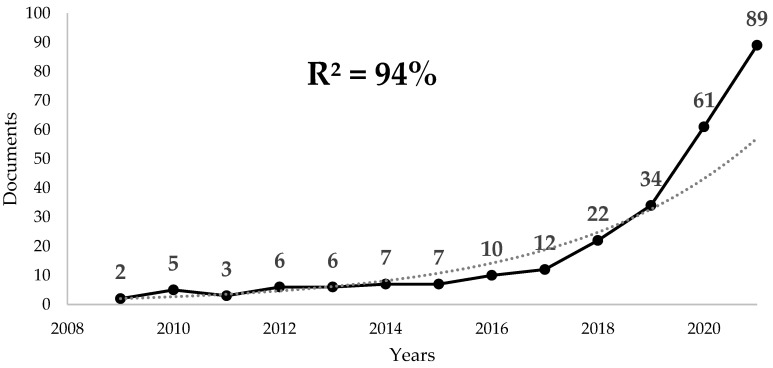
Annual publications trend on virtual reality and physical activity. R2: exponential growth rate.

A total of 341 documents (308 primary studies and 33 reviews), published between 1994 and 2022, were found. From 1994 to 2008, there was no continuity in the number of annual publications. Among those years, 2009 was the first with two or more annual publications with exponential growth until 2021 (Figure 1). During 2020 and 2021 (150 papers), the number of publications exceeded all previous years’ publications (120 documents).

### 3.2. WoS Categories

Documents were categorised into 90 thematic categories in the WoS. It was found that rehabilitation was the category with the most publications (44 documents), followed by sport sciences (40 documents), public environmental occupational health (39 documents), engineering electrical electronic (32 documents), and computer science software engineering (28 documents). Table 1 shows the top five thematic categories and the journals and publishers with the most publications. Other important categories were computer science information systems (19 documents), computer science interdisciplinary applications (19 documents), health care sciences services (19 documents), neurosciences (18 documents), and medical informatics (17 documents).

### 3.3. Publications Titles

The analysed publications were published in 205 journals, with a publication range between 1 and 12 studies. Bradford’s core was composed of 17 journals, all with four or more publications and accumulating 31.4% of the total number of publications. Table S1 shows the journals with the most publications in every subject area. The most prolific journals were games for health journal, in quartile 1 in the WoS rehabilitation category (12 papers), the International Journal of Environmental Research and Public Health (10 documents), and virtual reality (10 documents). The distribution followed by Bradford’s core (17 journals), Zone I (35 journals), and Zone II (153 journals) conformed to Bradford’s theoretical series, with a −20.7% error ratio, can be consulted in Table S2. According to the number of citations, Bradford’s core was composed of three journals, with 31.3% of the total citations, being the International Journal of Human-Computer Interaction (4 documents, 943 citations), located in quartile 1 in computer science, cybernetics, and ergonomics; presence-teleoperators and virtual environments (2 documents, 505 citations); and frontiers in robotics and AI (2 documents, 424 citations), indexed in the Emerging Sources Citation Index in the Robotics-SCI category. Table 2 displays Bradford’s core and Zone I for journals, according to the number of citations. The distribution followed by Bradford’s core (3 journals), Bradford’s zone I (17 journals), and Zone II (185 journals), according to the citations accumulated, was adjusted to Bradford’s theoretical series, with a -13.8% error ratio.

### 3.4. Most Prolific and Influential Co-Authors

**Figure 2:**
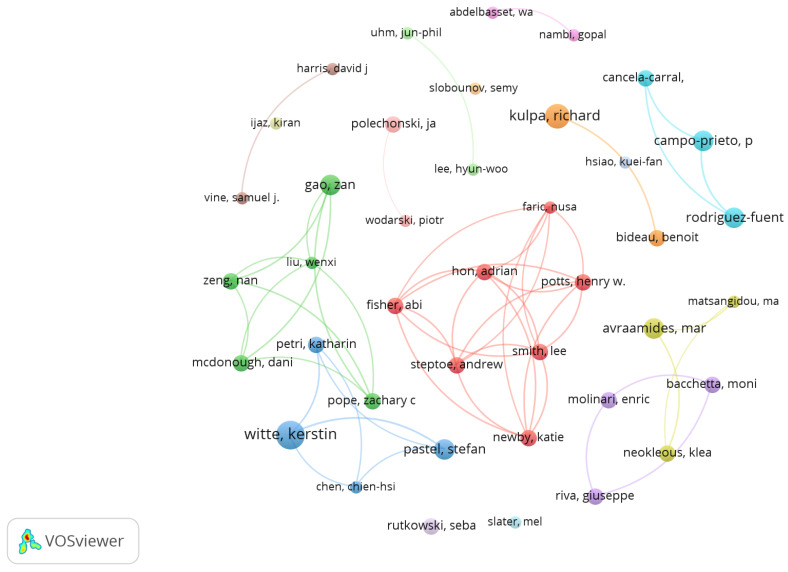
Most prolific co-authors and co-authoring connections. Analysis: fractionalization, attraction: 10, repulsion: −2.

A total of 1356 co-authors were identified, 88.9% with one paper (1205 co-authors), 8.9% with two (110 co-authors), and 3% with three or more (40 co-authors). Applying Lotka’s law, the number of most prolific co-authors was estimated as the 37 co-authors publishing most of the papers. Then, 25 co-authors with four or more papers and 40 co-authors with three or more papers were found, and these were considered the most prolific. Figure 2 displays the 40 most prolific co-authors on the topic, as well as the interactions between them in the publications. The most prolific co-author was Kerstin Witte (7 documents) from Otto von Guericke University (Germany), followed by Richard Kulpa (6 documents) from the University of Rennes (France), Marios Avraamides (5 documents) from the University of Cyprus (Cyprus), and Zan Gao (5 documents) from the University of Minnesota (USA).

After applying the h-index to the 40 most prolific co-authors, 25 co-authors with 27 or more co-citations were considered. Table 3 shows the 25 prominent co-authors, their affiliations, countries/regions, and the number of papers and citations. The most cited were Kiran Ijaz (936 citations) from Macquarie University (Australia) and Mel Slater (401 citations) from the University of Barcelona (Spain).

**Figure 3:**
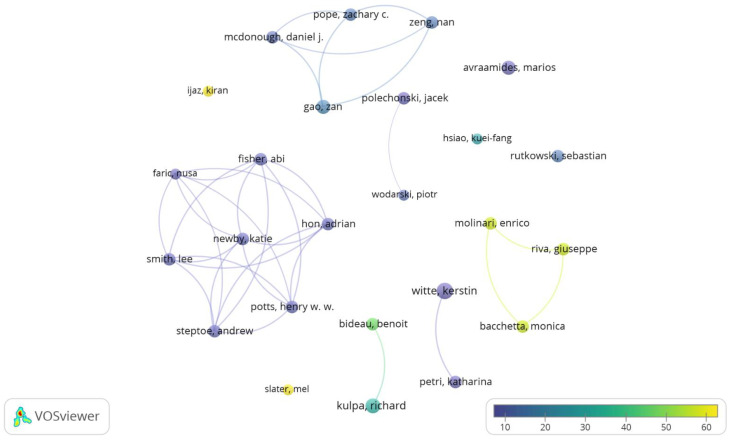
Prominent co-authors. Analysis: fractionalization, attraction: 10, repulsion: −2, node size: document number, and colour: average citation number.

Figure 3 shows the most prominent co-authors and their relations in the publications, highlighting the co-authors with the highest document number and those with the highest citation number. Figure S1 shows the most prominent co-authors and their interrelations, highlighting the groups of co-authors with more publications.

### 3.5. Countries/Regions

**Figure 4:**
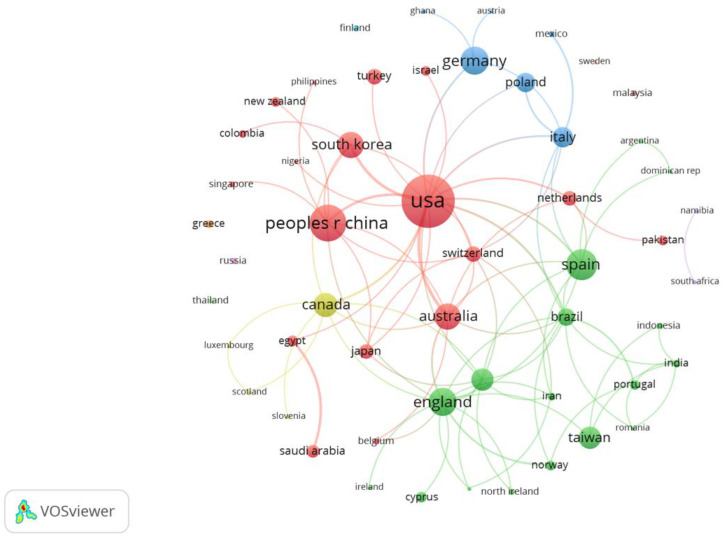
Co-authored countries/regions. Analysis: fractionalization, attraction: 7, repulsion: 0, clustering: resolution (0.5), minimum cluster size: 3, node side: documents, colour: cluster.

Publications were co-authored by 53 different countries/regions. The USA was the top country, with the highest number of co-authored papers (72 documents), followed by China (39 documents), Spain (29 documents), England (24 documents), and Germany (24 documents). England (1596 citations) was the country with the highest number of citations, followed by Australia (1360), the USA (1298), Republic of Korea (790), and Spain (648). The USA (17 links), England (14 links), France, Brazil, and Spain (10 links) had the highest number of co-authored links, with other countries/regions. Figure 4 shows the co-authored countries/regions, as well as the links and the clusters between them. A total of four main clusters were found: the red one, formed by 18 countries/regions, led by the USA, together with China, Switzerland, and Australia; the green, formed by 16 countries/regions, led by England, with France, Brazil, and Spain; the blue, formed by six countries/regions, led by Italy, with Germany and Poland; and the yellow one was composed of four countries/regions, led by Canada, with Luxembourg, Scotland, and Slovenia.

### 3.6. Author Keywords

**Figure 5:**
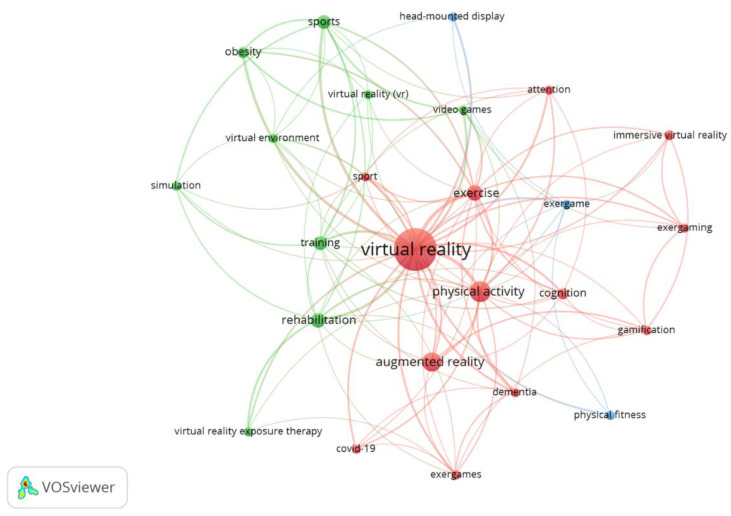
Most used author keywords. Analysis: association strength; attraction: 6, repulsion: −2, clustering: resolution (0.5) and minimum cluster size: -2, node size: occurrences, colour: cluster.

According to the search terms used in the search, the following were the most used: virtual reality (154 occurrences), physical activity (37 occurrences), augmented reality (30 occurrences), exercise (21 occurrences), or sport/sports (25 occurrences), as well as other terms, such as rehabilitation (19 occurrences), training (16 occurrences), obesity (11 occurrences), and cognition (10 occurrences), were the most frequently used terms. Figure 5 illustrates the most prominent author keywords and how they were interrelated in the analysed papers, revealing three clusters: the red one (13 keywords, including concepts such as dementia, cognition, and attention, together with virtual reality or physical activity), the green (9 keywords, including terms related to rehabilitation, therapy, and obesity), and the blue one (3 keywords: physical fitness, exergame, and head-mounted display). Figure S2 shows the same graph, with colours representing the average publication year and showing the most current keywords.

### 3.7. Documents

**Figure 6:**
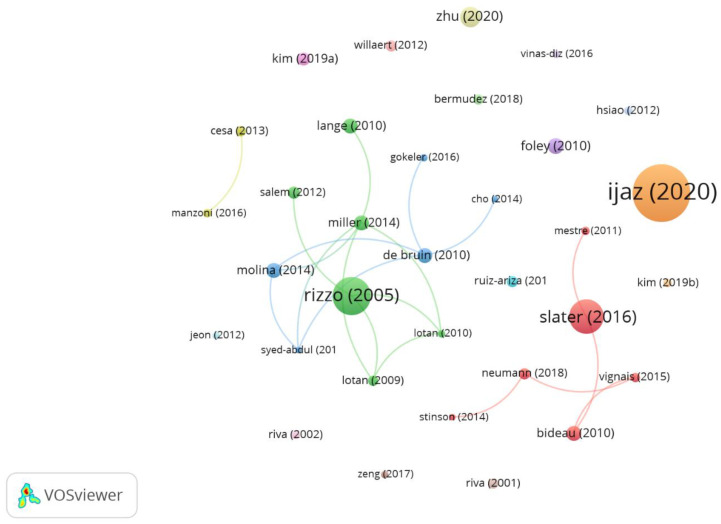
Most cited documents and their connections. Analysis: association strength, attraction: 6; repulsion: −2, node size: citations, colour: cluster.

The most relevant documents were the 33 documents with at least 33 citations within the analysed set of contributions (Table S3). The most cited article was “Player Experience of Needs Satisfaction (PENS) in an Immersive Virtual Reality Exercise Platform Describes Motivation and Enjoyment” by Karin Ijaz et al., published in the International Journal of Human-Computer Interaction in 2020, with 915 citations, with the following keywords: self-determination theory; active video game; physical-activity; intrinsic motivation; exergames; dance; determinants; information; children; and feedback. Other prominent publications were “A SWOT analysis of the field of virtual reality rehabilitation and therapy” by Albert Rizzo et al. (479 citations) and “Enhancing Our Lives with Immersive Virtual Reality” by Mel Slater et al. (401 citations). Figure 6 shows the interrelations between the most relevant publications.

## 4. Discussion

### 4.1. Main Findings

To our best knowledge, this is the first study conducting a bibliometric analysis of scientific publications on VR, AR, and AP, revealing that the number of annual publications on this topic follows an exponential growth trend, consistent with other bibliometrics with VR as a subject of study [61,63,78]. This proves the great interest in this subject among researchers, institutions, journals, etc., in line with the increase of users, companies, and investors involved with these technologies. The most productive and prolific journals and authors in the field, the most referenced papers, and the most used keywords were also identified.

The first publication on the topic appeared in 1994 [79], but no annual continuity was found until 2009. Subsequently, publications have continued an exponential growth trend, with a high increase from 2020 to 2021. A possible reason for this high growth may be related to the COVID-19 pandemic modifications in people’s lifestyle habits, with an increase in PA studies performed in isolated environments or with less social interaction to increase PA levels, to treat or prevent mental disorders, or to treat pathologies or injuries. The use of these technologies in rehabilitation has been identified in different studies [12,13,28,29,30,31,32,33]. In this line, the WoS category with the highest number of papers was rehabilitation, also including the most productive journal, *Games for Health Journal*, with the keywords VR, exposure therapy, and obesity. This category included papers such as “The Potential of Virtual Reality and Gaming to Assist Successful Aging with Disability” [80] or “Virtual reality using games for improving physical functioning in older adults: a systematic review” [81], both with more than 100 citations. The second thematic category with the most publications was sport sciences, with the most cited contribution being “Use of Active Video Games to Increase Physical Activity in Children: A (Virtual) Reality?” [46], which analyses the use of video games to promote PA in children. The contributions covered different areas, such as sports, training, visual perception, decision-making in sports, recovery from sports injuries, increasing PA, and motor skills, all related to the most frequent keywords: exercise, PA, training, sports, and physical fitness.

The interest in the topic’s publications was also demonstrated through the high impact factor of the top-producing journals. The three most productive journals were *Games for Health Journal*, *The International Journal of Environmental Research*, and *Public Health and Virtual Reality*, all ranked in the first quartile of their subject categories. Among the 17 journals in Bradford’s Core, 11 had an open access percentage (95%). Therefore, open access seems to be of great importance in the choice of researchers, as the number of publishers was very varied (JMIR Publications, Frontiers Media, Public Library, or Nature Portfolio). In contrast, the most prolific journal presented a very low percentage of open access (1.4%). Thus, researchers have a wide range of prestigious journals to publish in this area. In contrast, the Bradford’s core of the journals by the number of citations was much smaller, including only three journals with the highest number of citations: *The International Journal of Human-Computer Interaction*, *Presence-teleoperators and Virtual Environments,* and *Frontiers in Robotics and AI*. However, the high volume of citations accumulated by these journals was affected by the publication of the three most cited papers in them. First, “The Player Experience of Needs Satisfaction (PENS) in an Immersive Virtual Reality Exercise Platform Describes Motivation and Enjoyment” [53] aimed to understand why immersive VR was more interesting than a platform with a static user interface. Even though it was recently published (2020), this paper has become one of the most important on VR, being cited in the WoS thematic categories: psychology multidisciplinary (91 papers), educational research (79), psychiatry (78), environmental sciences (74), and public environmental occupational health (66). The study “A SWOT Analysis of the Field of Virtual Reality Rehabilitation and Therapy” [13] tried to provide an overview of the key issues for the understanding of the therapeutic use of VR, being a reference work in the WoS thematic category of rehabilitation (116 papers). Finally, the paper “Enhancing Our Lives with Immersive Virtual Reality” [11] examined VR applications, with some evidence being cited in fifty articles from the WoS thematic categories of computer science cybernetics, software engineering, and interdisciplinary applications.

As in previous bibliometric studies on these technologies [10,62,82], the USA was the country with the highest number of publications. However, the most productive author was Kerstin Witte from Germany, with seven papers, although with a low number of citations (36), as her studies have been recently published (2019–2022). The second most prolific co-author, Richard Kulpa (France), who has published on VR and sports performance, accumulated a high number of papers (6) and citations (188), as a reference in this subject, along with another recent co-author, Benoit Bideau, with “Using Virtual Reality to Analyze Sports Performance” being his most cited study [83]. Another group of prolific co-authors (4 papers), Enrico Molinari, Guiseppe Riva, and Monica Baccetta, have published on VR and therapies for the treatment of body image disorders in people with obesity [84,85,86,87], with an average of 60 citations per paper. Despite finding research groups with a high number of publications and connections between researchers from different countries, large groups are not formed yet, and collaboration between groups, institutions, and countries could increase the productivity and effectiveness of the research.

### 4.2. Practical Applications

VR and AR applications in healthcare, medicine, and rehabilitation are revolutionising the way professionals approach patient care; with their ability to provide immersive, interactive, and engaging experiences, these technologies hold the potential to improve patient outcomes and reduce costs, making them an important area for further research and investment. Moreover, they are becoming essential tools in Biomedicine and Bioengineering because of their ability to provide accurate, interactive, and immersive simulations of biological systems [88]. Thus, these technologies make it possible to deliver home-based assessments and interventions or telehealth, potentially offering cost-effective alternatives in several areas [59,89]. Furthermore, multiple practical applications are also emerging in sports sciences to increase the population’s PA levels through exergames, performance enhancement programs with technical or tactical training applications, and sports injuries recovery. Then, some of the practical implications of this study include that it may enable publishers, journals, and editors to understand the growing state of interest in VR-, AR-, and AP-related research, and the large pool of researchers behind the development of this topic of study. Additionally, it may provide valuable information to researchers on research trends in the field, as well as meeting other researchers and research groups, favouring the identification of relevant authors in different areas, and facilitating opportunities for collaboration and new partnerships and networks.

### 4.3. Limitations and Future Lines

This research has limitations, which include the fact that, despite using one of the most complete databases and widely used in bibliometric studies (The Web of Science), a publication bias may have occurred (of which, the consequences may include inaccurate analysis of research trends, biased citation analysis, difficulties replicating studies, or misleading indicators of research impact), excluding documents published in journals with lower impact factor or with characteristics different from those indexed in the WoS database. Future research should extend this analysis by including manuscripts from other databases, such as PubMed or Scopus, among others.

## 5. Conclusions

VR, AR, and PA annual publications showed an exponential growth trend in recent years, including large numbers of researchers, journals, and publishers interested in the topic. The use of VR in rehabilitation was the topic of most interest among researchers. The most productive core of journals consisted of high-impact journals, with most of them published with open access. *Games for Health* was the most productive journal. The most prolific author was Kerstin Witte, with Richard Kulpa being one of the most prominent. The USA was the most productive country, while England was the country with the highest number of citations.

## Figures and Tables

**Table 1:** Top five thematic categories in the Web of Science, according to the number of documents in which publications were indexed.

WoS Categories	Docs.	Journals (Publishers)	Docs.	Publishers	Docs.
Rehabilitation	44	Games for Health Journal (Mary Ann Liebert)	12	Mary Ann Liebert Inc.	12
Sport Sciences	40	Frontiers in Sports and Active Living (Frontiers)	5	Taylor and Francis	8
Public Environmental Occupational Health	39	Games for Health Journal (Mary Ann Liebert)	12	Mary Ann Liebert Inc.	12
Engineering Electrical Electronic	32	Sensors (MDPI)	8	MDPI	9
Computer Science Software Engineering	28	Virtual Reality (Springer)	10	Springer Nature	14

Docs: Documents number.

**Table 2:** Bradford’s Core and Zone I journals according to the number of citations.

Bradford’s Zone	Journals (Publishers)	Docs.	% Cit.	Cit.	JIF	Q.	% O.A.
Core	International Journal of Human-Computer Interaction (Taylor & Francis Inc.)	4	943	15.7%	4.920	Q1	8.7%
	Presence-Teleoperators and Virtual Environments (MIT Press)	2	505	8.4%	N/A	N/A	n.a.
	Frontiers in Robotics and AI (Frontiers Media SA)	2	424	7.1%	N/A	N/A	99.3%
Zone I	Science Advances (American Association for the Advancement of Science)	2	249	4.2%	14.972	Q1	90.1%
	Virtual Reality (Springer London Ltd.)	10	138	2.3%	4.697	Q1	28.4%
	Games for Health Journal (Mary Ann Liebert Inc.)	12	135	2.3%	4.070	Q1	1.4%
	Research in Developmental Disabilities (Pergamon-Elsevier)	3	134	2.2%	3.000	Q1	11.5%
	Computers In Human Behaviour (Pergamon-Elsevier)	3	132	2.2%	8.957	Q1	11.3%
	Pediatric Exercise Science (Human Kinetics Inc.)	1	122	2.0%	2.395	Q3	0.0%
	Journal of Neuroengineering and Rehabilitation (BMC)	2	119	2.0%	5.208	Q1	99.6%
	Journal of Medical Internet Research (JMIR Publications Inc.)	7	117	2.0%	7.093	Q1	99.2%
	Age and Ageing (Oxford University Press)	2	115	1.9%	12.782	Q1	31.7%
	Physical Medicine and Rehabilitation Clinics of North America (Saunders-Elsevier)	1	114	1.9%	2.391	Q2	1.3%
	IEEE Computer Graphics and Applications (IEEE Computer Society)	1	113	1.9%	1.909	Q3	3.6%
	Zeitschrift fur Gerontologie und Geriatrie (Springer Heidelberg)	1	107	1.8%	1.292	Q4	29.9%
	Computers and Education (Pergamon-Elsevier)	2	102	1.7%	11.182	Q1	11.4%
	Cyberpsychology Behaviour and Social Networking (Mary Ann Liebert Inc.)	3	99	1.7%	6.135	Q1	5.8%
	Physiotherapy (Elsevier Science)	2	86	1.4%	3.704	Q1	17.5%
	International Journal of Environmental Research and Public Health (MDPI)	10	82	1.4%	4.614	Q1	95.0%
	Interactive Learning Environments (Routledge Journals, Taylor & Francis Ltd.)	2	70	1.2%	4.965	Q1	4.4%

Docs.: number of documents; Cit: number of citations; % cit.: percentage of citations; JIF: Journal Impact Factor; % O.A.: Open Access percentage; Q.: quartile; N/A: not applicable.

**Table 3:** Prominent co-authors on Virtual and Augmented Reality and Physical Activity.

Co-Authors	Affiliation/Countries-Regions	Documents	Citations
Witte, Kerstin	Otto von Guericke University/Germany	7	36
Kulpa, Richard	University of Rennes/France	6	188
Avraamides, Marios	University of Cyprus/Cyprus	5	27
Gao, Zan	University of Minnesota/USA	5	92
Bacchetta, Monica	IRCCS Italian Auxological Institute/Italy	4	221
Bideau, Benoit	University of Rennes/France	4	183
Fisher, Abi	University College London/England	4	45
Hon, Adrian	Six Start/England	4	45
Mcdonough, Daniel	University of Minnesota Twin Cities/USA	4	54
Molinari, Enrico	IRCCS Italian Auxological Institute/Italy	4	221
Newby, Katie	University of Hertfordshire/England	4	45
Petri, Katharina	Otto von Guericke University/Germany	4	35
Polechonski, Jacek	Academy of Physical Education in Katowice/Poland	4	39
Pope, Zachary	Well Living Lab/USA	4	62
Potts, Henry	University College London/England	4	45
Riva, Giuseppe	Catholic University of the Sacred Heart/Italy	4	221
Rutkowski, Sebastian	Opole University of Technology/Poland	4	63
Smith, Lee	Anglia Ruskin University/University	4	45
Steptoe, Andrew	University College London/England	4	45
Zeng, Nan	University of New Mexico/USA	4	62
Faric, Nusa	University College London/England	3	29
Hsiao, Kuei-Fang	University of Sharjah/United Arab Emirates	3	82
Ijaz, Kiran	Macquarie University/Australia	3	936
Slater, Mel	University of Barcelona/Spain	3	401
Wodarski, Piotr	Silesian University of Technology/Poland	3	40

## Data Availability

Datasets are available through the corresponding author upon reasonable request.

## Supplementary Materials

The following supporting information can be downloaded at: https://www.mdpi.com/article/10.3390/s23062987/s1.
