# Supplementary material for: Rehabilitation Is the Main Topic in Virtual and Augmented Reality and Physical Activity Research: A Bibliometric Analysis
Source: Sensors (Basel). 2023 Mar 9;23(6):2987. doi: 10.3390/s23062987 (PMC10056397; doi:10.3390/s23062987)
Supplement: Supplementary file 1 [file sensors-23-02987-s001.zip › Tables S1-S3.pdf]

Table S1. Most prolific journals

Bradford's zone	Journals (Publishers)	Doc.	% Doc.	Cit.	JIF	Q.	% O.A.
CORE	Games for health journal (Mary Ann Liebert Inc)	12	3.5%	135	4.070	Q1	1.4%
	International journal of environmental research and public health (Mdpi)	10	2.9%	82	4.614	Q1	95.0%
	Virtual reality (Springer London Ltd)	10	2.9%	138	4.697	Q1	28.4%
	JMIR serious games (JMIR Publications, INC)	8	2.3%	55	3.364	Q2	99.5%
	Sensors (Mdpi)	8	2.3%	21	3.847	Q2	95.2%
	Frontiers in psychology (Frontiers Media SA)	7	2.1%	25	4.232	Q1	99.3%
	Journal of medical internet research (JMIR Publications, INC)	7	2.1%	117	7.093	Q1	99.2%
	Applied sciences-basel (Mdpi)	5	1.5%	10	2.838	Q2	97.4%
	Frontiers in neurology (Frontiers Media SA)	5	1.5%	11	4.086	Q2	99.2%
	Frontiers in sports and active living (Frontiers Media SA)	5	1.5%	8	n.a.	n.a.	98.2%
	PloS One (Public Library Science)	5	1.5%	66	3.752	Q2	99.6%
	Sustainability (Mdpi)	5	1.5%	20	3.889	Q2	94.7%
	IEEE Transactions on visualization and computer graphics (IEEE Computer Soc)	4	1.2%	45	5.226	Q1	3.3%
	International journal of human-computer interaction (Taylor & Francis INC)	4	1.2%	943	4.920	Q1	8.7%
	Journal of sports sciences (Taylor & Francis Ltd)	4	1.2%	59	3.943	Q2	9.4%
	Multimedia tools and applications (Springer)	4	1.2%	32	2.577	Q2	4.0%
	Scientific reports (Nature Portfolio)	4	1.2%	4	4.996	Q2	99.6%

Doc. (Number of documents); Cit. (Number of citations); % Doc. (Percentage of documents); JIF (Journa impact factor); % O.A. (Percentage of open access); Q. (JIF Quartile); n.a. (not application).

Table S2. Bradford's zones and their number of journals, according to: number of articles; number of citations.

Bradford's zones and their number of journals, according to number of articles.

Zone	Nº journals (%)		Number articles (%)		Acc. nº journals (%)		Acc. nº articles (%)		Bradford multipliers		Journals (theoretical serie)	
CORE	17	(8.3%)	107	(31.4%)	17	(8.3%)	107	(31.4%)			n0	17
Zone 1	35	(17.1%)	81	(23.8%)	52	(25.4%)	188	(55.1%)		2.06	n1	55
Zone 2	153	(74.6%)	153	(44.9%)	205	(100%)	341	(100%)		4.37	n2	176
Total	205	100%	341	100%					Mean	3.22		247
											% Error	-20.7%

Bradford's zones and their number of journals, according to number of citations.

Zone	Nº journals (%)		Nº articles (%)		Number citations (%)		Acc. nº citations (%)		Bradford multipliers		Journals (theoretical serie)	
CORE	3	(1.5%)	8	(2.3%)	1872	(31.3%)	589	(31.3%)			n0	3
Zone 1	17	(8.3%)	64	(18.8%)	2034	(34.0%)	2623	(65.2%)		5.67	n1	25
Zone 2	185	(90.2%)	269	(78.9%)	2082	(34.8%)	4705	(100%)		10.88	n2	205
Total	205	100%	341	100%	5988	100%			Mean	8.3		233
											% Error	-13.8%

Nº (Number); % (Percentage); Acc (Accumulated);

Table S3. Most cited documents.

Title. Main author (Year of publication)	Journal ISO Abbreviation	Cites
Player Experience of Needs Satisfaction (PENS) in an Immersive Virtual Reality Exercise Platform Describes Motivation and Enjoyment	Int. J. Hum.-Comput. Interact.	915
A SWOT analysis of the field of virtual reality rehabilitation and therapy	Presence-Teleoper. Virtual Env.	479
Enhancing Our Lives with Immersive Virtual Reality	Front. Robot. AI	401
Haptic-feedback smart glove as a creative human-machine interface (HMI) for virtual/augmented reality applications	Sci. Adv.	183
Use of Active Video Games to Increase Physical Activity in Children: A (Virtual) Reality?	Pediatr. Exerc. Sci.	122
The Potential of Virtual Reality and Gaming to Assist Successful Aging with Disability	Phys. Med. Rehabil. Clin. N. Am.	114
Effectiveness and feasibility of virtual reality and gaming system use at home by older adults for enabling physical activity to improve health-related domains: a systematic review	Age Ageing	113
Using Virtual Reality to Analyze Sports Performance	IEEE Comput. Graph. Appl.	113
Use of virtual reality technique for the training of motor control in the elderly Some theoretical considerations	Z. Gerontol. Geriatr.	107
Virtual reality using games for improving physical functioning in older adults: a systematic review	J. NeuroEng. Rehabil.	107
The impact of virtual reality (VR) technology on sport spectators' flow experience and satisfaction	Comput. Hum. Behav.	86
Effectiveness of a low-cost virtual reality system for children with developmental delay: a preliminary randomised single-blind controlled trial	Physiotherapy	80
A systematic review of the application of interactive virtual reality to sport	Virtual Real.	75
Effect of augmented reality game Pokemon GO on cognitive performance and emotional intelligence in adolescent young	Comput. Educ.	71
Recent Advancements in Medical Simulation: Patient-Specific Virtual Reality Simulation	World J.Surg.	69
Magnetosensitive e-skins with directional perception for augmented reality	Sci. Adv.	66
Improving physical fitness of individuals with intellectual and developmental disability through a Virtual Reality Intervention Program	Res. Dev. Disabil.	64
Virtual reality-based multidimensional therapy for the treatment of body image disturbances in obesity: A controlled study	CyberPsychol. Behav.	64
Virtual Reality for Enhancing the Cognitive Behavioral Treatment of Obesity With Binge Eating Disorder: Randomized Controlled Study With One-Year Follow-up	J. Med. Internet Res.	64
Which technology to investigate visual perception in sport: Video vs. virtual reality	Hum. Mov. Sci.	52
Learning while exercising for science education in augmented reality among adolescents	Interact. Learn. Environ.	50
The effectiveness of virtual reality for people with mild cognitive impairment or dementia: a meta-analysis	BMC Psychiatry	49
Virtual-reality-based multidimensional therapy for the treatment of body image disturbances in binge eating disorders: A preliminary controlled study	IEEE T. Inf. Technol. Biomed.	49
Does Virtual Reality Enhance Exercise Performance, Enjoyment, and Dissociation? An Exploratory Study on a Stationary Bike Apparatus	PRESENCE-Virtual Augmented Reality	46
Virtual Reality-Enhanced Cognitive-Behavioral Therapy for Morbid Obesity: A Randomized Controlled Study with 1 Year Follow-Up	Cyberpsychology Behav. Soc. Netw.	44
Immersive virtual reality improves movement patterns in patients after ACL reconstruction: implications for enhanced criteria-based return-to-sport rehabilitation	Knee Surg. Sports Traumatol. Arthrosc.	42
Rendering Virtual Tumors in Real Tissue Mock-Ups Using Haptic Augmented Reality	IEEE Trans. Haptics	40
Virtual reality as means to improve physical fitness of individuals at a severe level of intellectual and developmental disability	Res. Dev. Disabil.	39
Acute Effect of Virtual Reality Exercise Bike Games on College Students' Physiological and Psychological Outcomes	Cyberpsychology Behav. Soc. Netw.	38
Virtual reality for therapeutic purposes in stroke: A systematic review	Neurologia	37
Virtual reality among the elderly: a usefulness and acceptance study from Taiwan	BMC Geriatr.	35
Feasibility of Training Athletes for High-Pressure Situations Using Virtual Reality	IEEE Trans. Vis. Comput. Graph.	35
The Effect of a Virtual Reality Exercise Program on Physical Fitness, Body Composition, and Fatigue in Hemodialysis Patients	J. Phys. Ther. Sci.	34

Cites (Times cited in Web of Science Core).
