# Supplementary figures and images for: Rehabilitation Is the Main Topic in Virtual and Augmented Reality and Physical Activity Research: A Bibliometric Analysis
Source: Sensors (Basel). 2023 Mar 9;23(6):2987. doi: 10.3390/s23062987 (PMC10056397; doi:10.3390/s23062987)

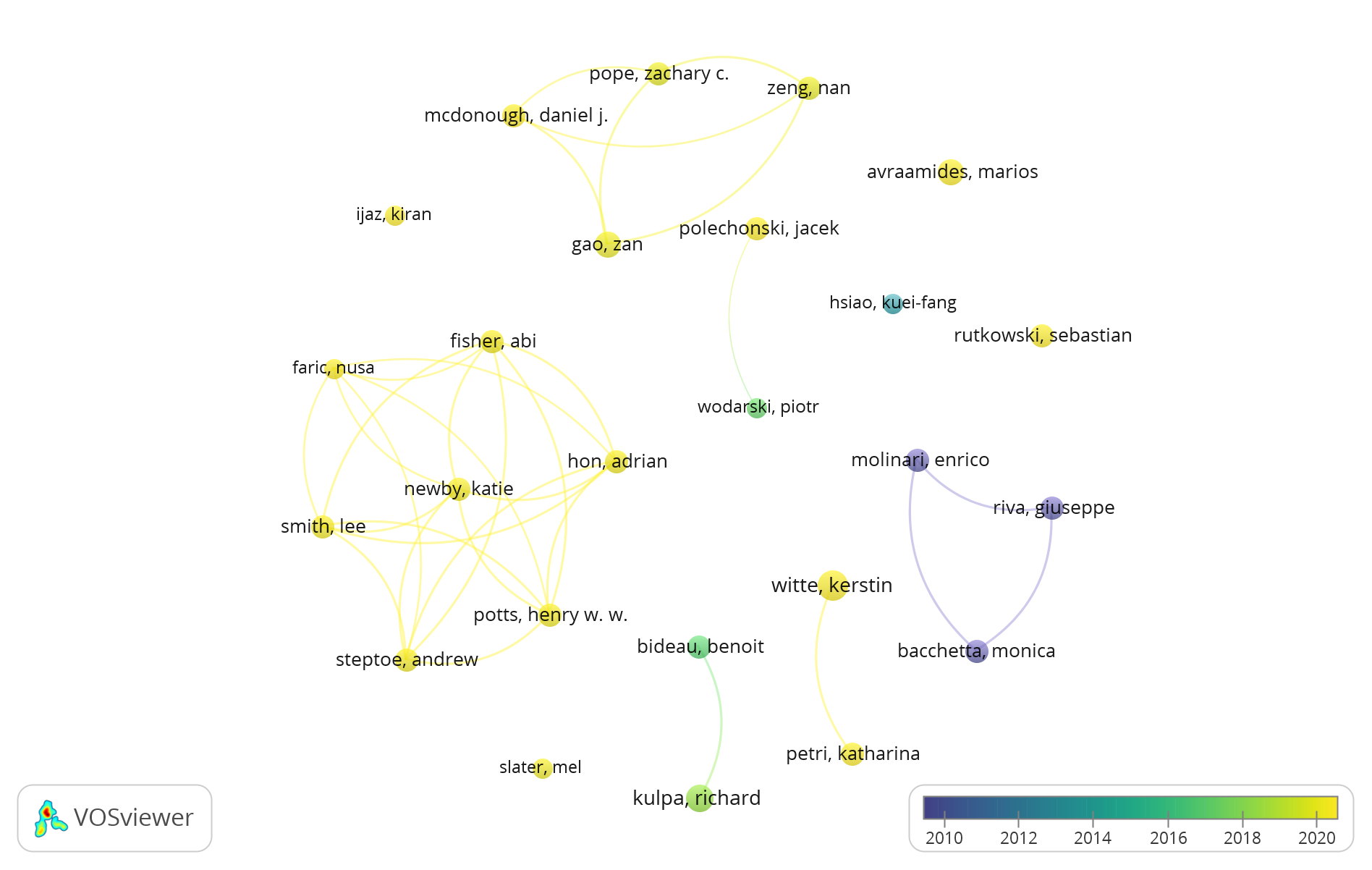

Supplement: Supplementary file 1 [file sensors-23-02987-s001.zip › Figure S1 Prominent Co_Authors.png]

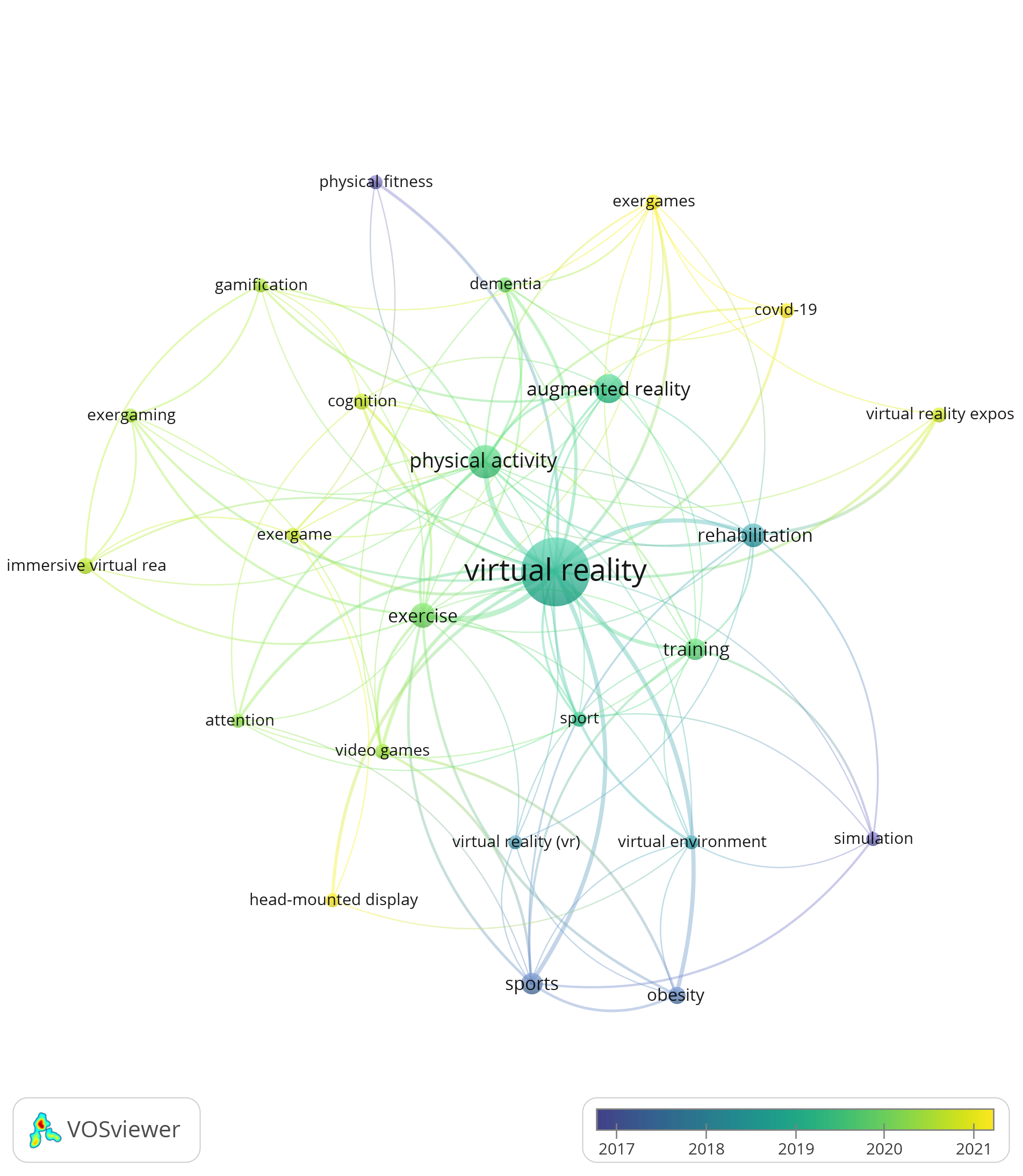

Supplement: Supplementary file 1 [file sensors-23-02987-s001.zip › Figure S2 Author Keywords.png]
